# Tat-CIAPIN1 Prevents Pancreatic β-Cell Death in hIAPP-Induced RINm5F Cells and T2DM Animal Model

**DOI:** 10.3390/ijms241310478

**Published:** 2023-06-22

**Authors:** Hyeon Ji Yeo, Min Jea Shin, Ki-Yeon Yoo, Bo Hyun Jung, Won Sik Eum, Soo Young Choi

**Affiliations:** 1 Department of Biomedical Science and Research, Institute of Bioscience and Biotechnology, Hallym University, Chuncheon 24252, Republic of Korea; hjyeo@hallym.ac.kr (H.J.Y.); wehome3@hallym.ac.kr (M.J.S.); 2 Department of Anatomy, College of Dentistry, Gangneung-Wonju National University, Gangneung 25457, Republic of Korea; kyyoo@gwnu.ac.kr (K.-Y.Y.); jungbh@gwnu.ac.kr (B.H.J.)

**Keywords:** Tat-CIAPIN1, T2DM, IAPP, MAPK, protein therapy

## Abstract

It is well known that the cytokine-induced apoptosis inhibitor 1 (CIAPIN1) protein plays an important role in biological progresses as an anti-apoptotic protein. Human islet amyloid peptide (hIAPP), known as amylin, is caused to pancreatic β-cell death in type 2 diabetes mellitus (T2DM). However, the function of CIAPIN1 protein on T2DM is not yet well studied. Therefore, we investigated the effects of CIAPIN1 protein on a hIAPP-induced RINm5F cell and T2DM animal model induced by a high-fat diet (HFD) and streptozotocin (STZ). The Tat-CIAPIN1 protein reduced the activation of mitogen-activated protein kinase (MAPK) and regulated the apoptosis-related protein expression levels including COX-2, iNOS, Bcl-2, Bax, and Caspase-3 in hIAPP-induced RINm5F cells. In a T2DM mice model, the Tat-CIAPIN1 protein ameliorated the pathological changes of pancreatic β-cells and reduced the fasting blood glucose, body weight and hemoglobin Alc (HbAlc) levels. In conclusion, the Tat-CIAPIN1 protein showed protective effects against T2DM by protection of β-cells via inhibition of hIAPP toxicity and by regulation of a MAPK signal pathway, suggesting CIAPIN1 protein can be a therapeutic protein drug candidate by beneficial regulation of T2DM.

## 1. Introduction

A thirty-seven-amino acid-polypeptide hormone human islet amyloid peptide (hIAPP), known as amylin, is synthesized and secreted along with insulin from pancreatic β-cells [1,2]. The structure of hIAPP is critical for β-pleated sheet formation which tends to form amyloid fibrils [3] and hIAPP has diverse metabolic roles, including insulin release, regulation of food intake, energy homeostasis, and gastric emptying under physiological conditions [4,5,6]. It is well known that type 2 diabetes mellitus (T2DM) is an energy metabolic disorder characterized by high blood glucose, insulin resistance, dysfunction of pancreatic β-cells, loss of the number of pancreatic β-cells, and deposits of amyloid in Langerhans islets [7,8,9]. hIAPP is the major component of amyloid deposits in pancreatic islets shown in pathological conditions of more than 90% of T2DM patients [10]. In a hIAPP transgenic mouse model, hyperglycemia increases amyloid formation with a decreasing β-cell mass [2,11].

It has been reported that reactive oxygen species (ROS) are needed for the maintenance and regulation of physiological processes including cell proliferation and apoptosis under normal conditions. However, excessive ROS production induced by oxidative stress can lead to damage of macromolecules related to cell survival [12,13]. ROS can also regulate signaling for proliferation and cell viability [14] and previous studies have shown that loss of pancreatic β-cell function can cause oxidative stress and activation of mitogen-activated protein kinase (MAPKs) [15,16]. Therefore, the decrease in oxidative stress and MAPK signaling is important for the therapeutic approach by controlling of DM.

Cytokine-induced apoptosis inhibitor 1 (CIAPIN1) is an anti-apoptotic molecule expressed in both the nucleus and cytoplasm of most tissues [17]. CIAPIN1 plays key roles in cancers such as gastric, hepatocellular carcinoma, and renal cancers and it has been suggested that CIAPIN1 has an important role in cancer therapy [18,19,20]. Park et al. demonstrated that CIAPIN1 protein is associated with neurodegeneration in dopaminergic neuronal cells [21]. We also have shown that CIAPIN1 protein has protective effects against hippocampal neuronal HT-22 cell death under oxidative stress conditions and a brain ischemic injury animal model [22]. Although CIAPIN1 protein is related to oxidative stress, its role in T2DM is still not fully investigated.

Several reports have shown that protein transduction peptides (PTD) can deliver active cargo into cells and delivered PTD fusion protein can be used as an effective tool for the application of therapeutic proteins for various diseases. Although the precise mechanism of delivery is unclear, HIV-1 Tat transduction domain, Tat peptide (YGRKKRRQRRR), is the most common PTD [23,24,25,26,27]. Numerous studies have shown that PTD fusion proteins play a protective role in suppressing disease in cells and animal models of various diseases [28,29,30,31,32,33,34,35]. Here, we examined whether cell permeable Tat-CIAPIN1 fusion protein could protect against hIAPP-induced cytotoxicity and pancreatic β-cell dysfunction.

## 2. Results

### 2.1. Delivery of Tat-CIAPIN1 Protein into RINm5F Cells

**Figure 1:**
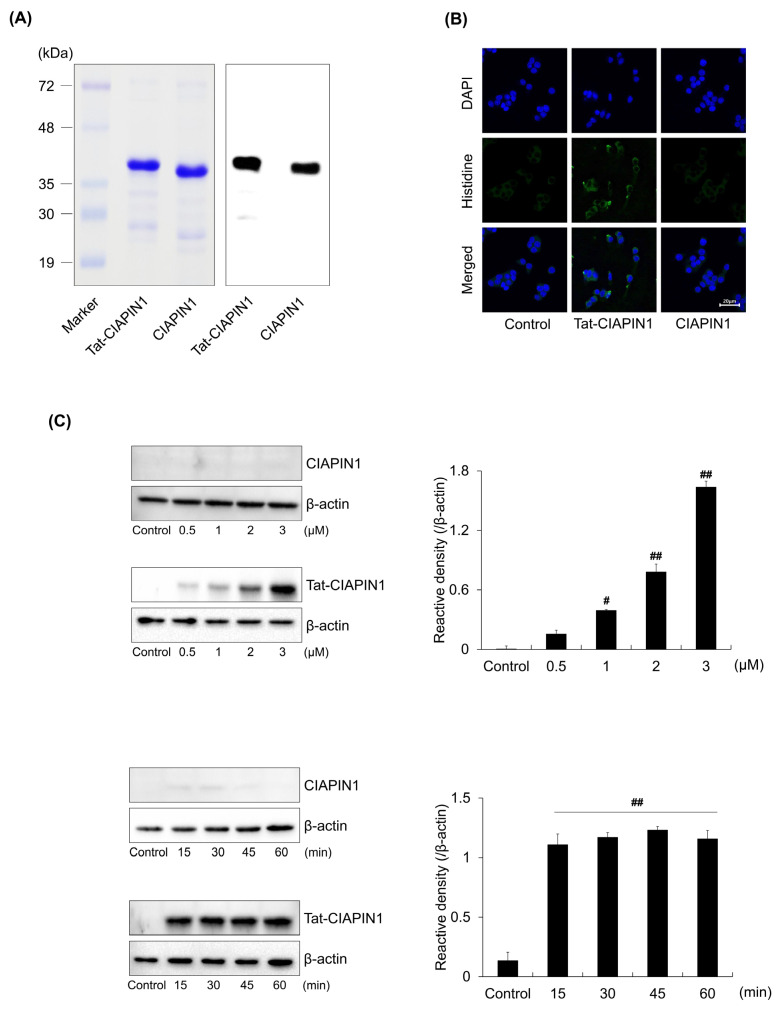
Cellular localization of Tat-CIAPIN1 protein. (**A**) Purification of Tat-CIAPIN1 and CIAPIN1 proteins were analyzed by SDS-PAGE and Western blotting with an anti-histidine antibody. (**B**) The localization of delivered Tat-CIAPIN1 protein was detected by confocal fluorescence microscopy. Scale bar = 20 μm. (**C**) Tat-CIAPIN1 (0.5–3 μM) protein was treatment with RINm5F cells for 1 h or Tat-CIAPIN1 (3 μM) protein was treatment with RINm5F cells for 15–60 min. ^#^ *p* < 0.05 and ^##^ *p* < 0.01, compared to control cells.

We prepared the Tat-CIAPIN1 protein as described previously [22]. As shown in Figure 1A, purified Tat-CIAPIN1 protein was determined by SDS-PAGE and Western blotting. Additionally, the delivery of the Tat-CIAPIN1 protein was confirmed in RINm5F cells. Immunofluorescence staining demonstrated that delivered Tat-CIAPIN1 protein was distributed throughout cells (Figure 1B). Moreover, we confirmed the delivery of Tat-CIAPIN1 protein according to concentration and time by Western blotting. As shown in Figure 1C, Tat-CIAPIN1 protein was delivered into the cells rapidly and in a concentrated form. On the other hand, CIAPIN1 as a control was not delivered into cells.

### 2.2. Effects of Tat-CIAPIN1 Protein on hIAPP-Induced RINm5F Cell Viability

**Figure 2:**
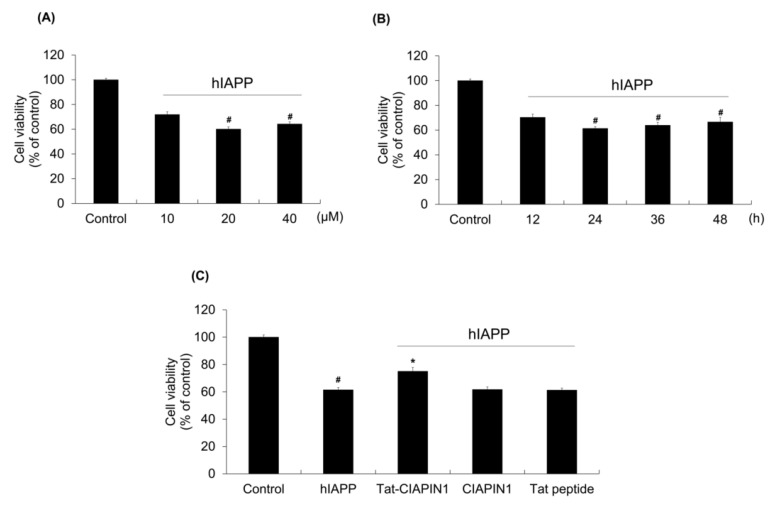
Effect of Tat-CIAPIN1 protein on hIAPP-induced cell death. The effects of hIAPP on RINm5F cell viability. (**A**) Cells were treated with 10–40 μM hIAPP for 48 h, or (**B**) cells were treated with 20 μM hIAPP for time periods and cell viability was determined using MTT assay. ^#^ *p* < 0.05, compared to control cells. (**C**) Effects of Tat-CIAPIN1 against hIAPP-induced cell death. Cells pretreated with Tat-CIAPIN1 (3 μM) for 1 h were incubated with 20 μM hIAPP for 24 h, and cell viability was determined using MTT assay. ^#^ *p* < 0.05, compared to control cells, * *p* < 0.05, compared to hIAPP-treated cells.

To clarify the optimal concentration of hIAPP used in this study, we used a range of different concentrations (10–40 μΜ) or times (12–48 h) of hIAPP to treat the RINm5F cells and MTT assays were then carried out. As shown in Figure 2A,B, the concentrations of (20 μΜ and 40 μΜ) and times (from 24 to 48 h) showed similar effects on the cells. Therefore, we chose the concentration (20 μΜ) and time (24 h) as the treatment in this study.

To examine the effect of the Tat-CIAPIN1 protein on hIAPP-induced cell viability, we pretreated cells with Tat-CIAPIN1 protein (3 μΜ) for 1 h and exposed cells to 20 μΜ of hIAPP for 24 h and cell viability was determined. We showed that Tat-CIAPIN1 protein markedly increased the level of cell viability, whereas CIAPIN1 or Tat peptide have no effects (Figure 2C).

**Figure 3:**
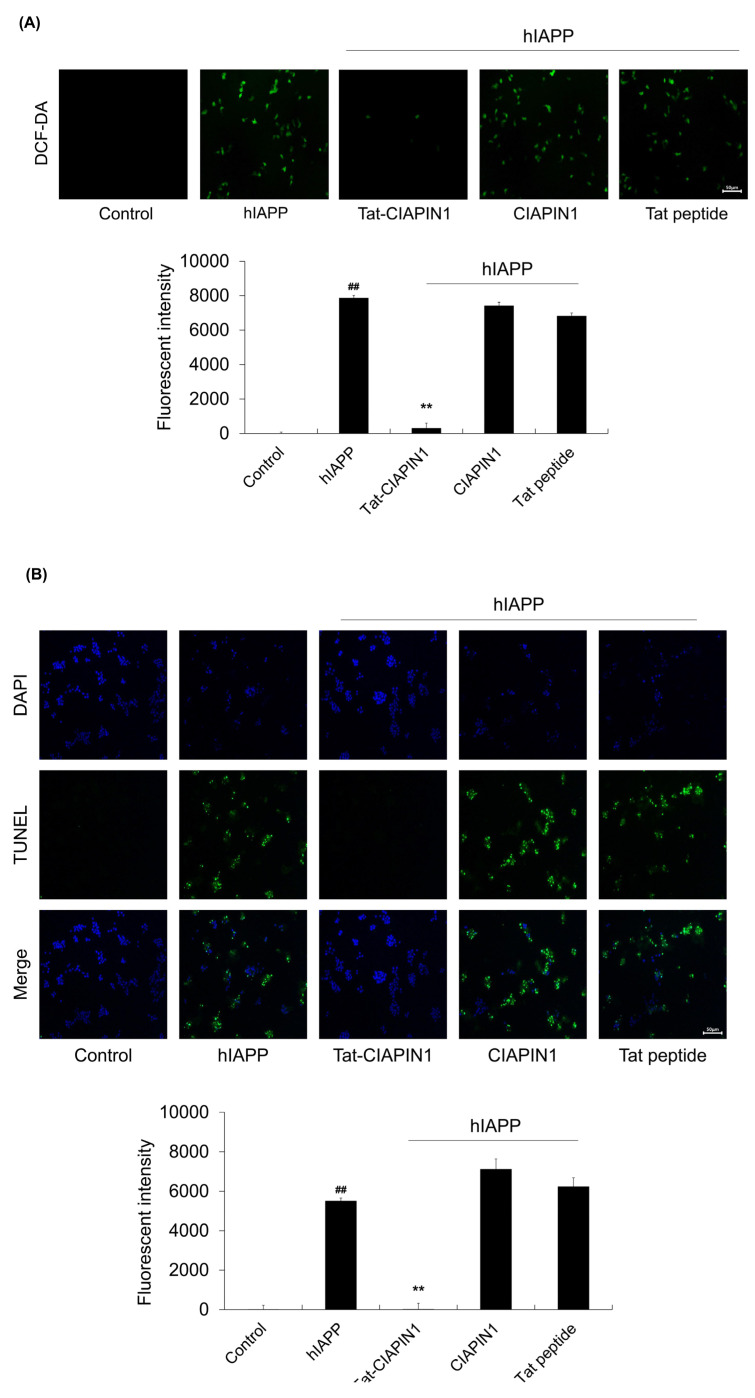
Effects of Tat-CIAPIN1 protein on hIAPP-induced ROS production and DNA fragmentation. RINm5F cells were treated with Tat-CIAPIN1 (3 μM), CIAPIN1, and Tat peptide for 1 h before treatment with 20 μM hIAPP. Then, intracellular ROS levels (**A**) and DNA fragmentation (**B**) were determined by DCF-DA and TUNEL stains. Fluorescence intensity was quantified using an ELISA plate reader. Scale bar = 50 μm. ^##^ *p* < 0.01, compared to control cells, ** *p* < 0.01, compared to hIAPP-treated cells.

We further investigated whether Tat-CIAPIN1 protein could inhibit DNA damage and ROS production in hIAPP exposed cells. As shown in Figure 3, ROS and DNA damage levels were significantly increased after treatment with hIAPP. However, Tat-CIAPIN1 protein markedly reduced ROS and DNA damage levels compared to hIAPP treatment alone. CIAPIN1 and Tat peptide had no effect on cytotoxicity induced by hIAPP in RINm5F cells.

### 2.3. Effects of Tat-CIAPIN1 Protein on hIAPP-Induced MAPK and Apoptotic Protein Expression in RINm5F Cells

**Figure 4:**
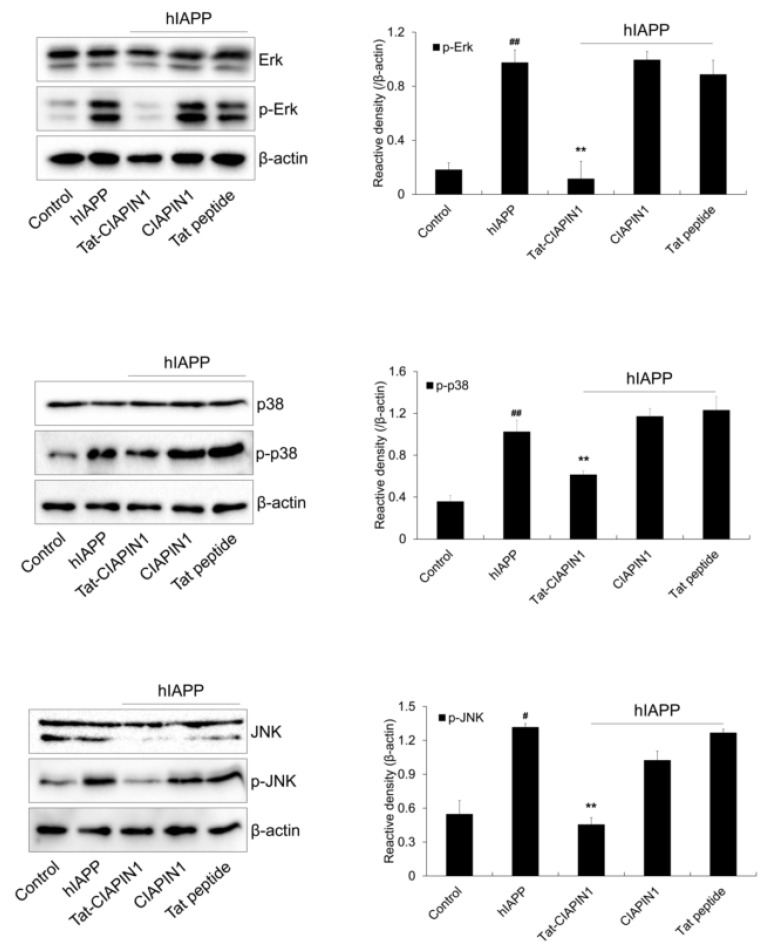
Effects of Tat-CIAPIN1 protein on hIAPP-induced expression of phosphorylation of MAPKs in RINm5F cells. The cells were treated with Tat-CIAPIN1 (3 μM), CIAPIN1, and Tat peptide for 1 h before being exposed to hIAPP (20 μM). The expression levels of phosphorylated MAPKs were analyzed by Western blotting. Band intensity was measured by densitometer. ^#^ *p* < 0.05 and ^##^ *p* < 0.01, compared to control cells, ** *p* < 0.01, compared to hIAPP-treated cells.

Several studies have reported that hIAPP stimulates MAPK signaling and apoptotic cell death in pancreatic β-cells [36,37,38,39]. We examined the effects of Tat-CIAPIN1 on hIAPP-induced MAPK signaling. As shown in Figure 4, phosphorylated MAPKs were increased in cells treated with hIAPP only. However, Tat-CIAPIN1 markedly reduced phosphorylated MAPK levels compared with hIAPP treatment only.

**Figure 5:**
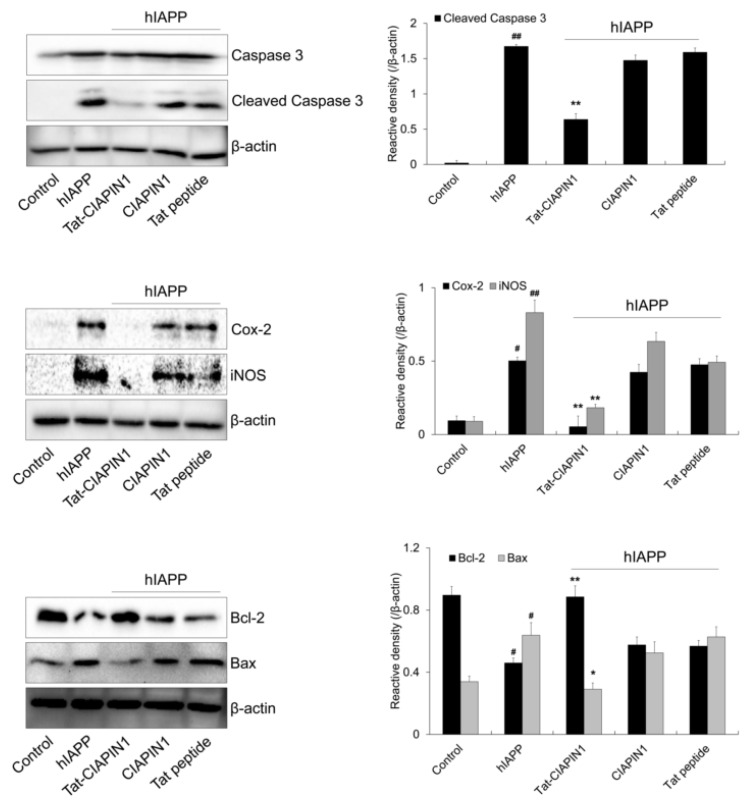
Effects of Tat-CIAPIN1 protein on hIAPP-induced expression of apoptotic proteins in RINm5F cells. The cells were treated with Tat-CIAPIN1 (3 μM), CIAPIN1, and Tat peptide for 1 h before being exposed to hIAPP (20 μM). The expression of apoptotic proteins levels were analyzed by Western blotting. Band intensity was measured by densitometer. ^#^ *p* < 0.05 and ^##^ *p* < 0.01, compared to control cells, * *p* < 0.05 and ** *p* < 0.01, compared to hIAPP-treated cells.

Furthermore, we investigated the expression levels of apoptosis-related proteins after treatment with hIAPP. Expression levels of apoptosis-related proteins (cleaved caspase-3, Bax, COX-2, and iNOS) were increased in cells treated with hIAPP only (Figure 5). However, Tat-CIAPIN1 markedly reduced expression levels of these proteins. Bcl-2 expression level was reduced by hIAPP, but increased by Tat-CIAPIN1. However, CIAPIN1 and Tat peptide had no effect on expression levels of these proteins.

### 2.4. Effects of Tat-CIAPIN1 in T2DM Mice Model

**Figure 6:**
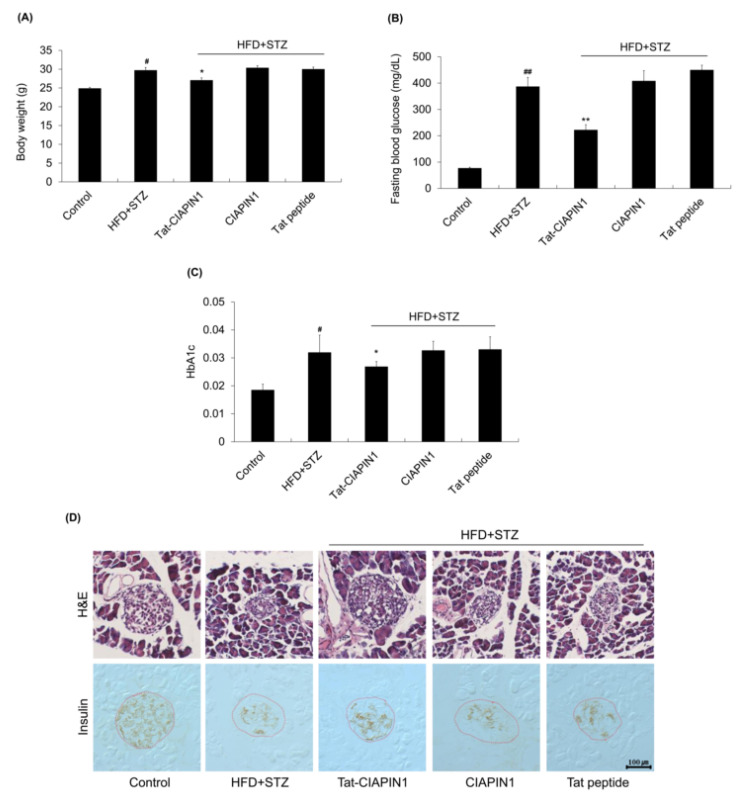
Effects of Tat-CIAPIN1 protein on HFD/STZ-induced diabetic mice after 8 weeks. The effect of Tat-CIAPIN1 on (**A**) body weight, (**B**) blood glucose, and (**C**) HbA1c levels were determined in HFD/STZ-induced diabetic mice. (**D**) Pancreas sections were stained with HE and insulin. The red dotted line denotes the Langerhans islets. Scale bar = 100 μm. ^#^ *p* < 0.05 and ^##^ *p* < 0.01, compared to control group, * *p* < 0.05 and ** *p* < 0.01, compared with the HFD/STZ-induced group.

To determine effects of Tat-CIAPIN1 in T2DM, we prepared a T2DM model using HFD combined with STZ. We observed that body weight, fasting blood glucose, and hemoglobin A1c (HbA1c) levels were increased in the HFD + STZ group than in the control. Body weight, fasting blood glucose, and HbA1c levels in CIAPIN1 or Tat peptide group were similar to those of the HFD + STZ group, but markedly reduced in Tat-CIAPIN1 administrated group (Figure 6A–C). As shown in Figure 6D, pancreatic islet was destructed in the HFD + STZ group. Tat-CIAPIN1 administration protected against the destruction of pancreatic islets and loss of insulin levels by STZ. However, CIAPIN1 and Tat peptide did not affect the T2DM model. 

## 3. Discussion

Human IAPP can form amyloid deposits in islets of T2DM patients and lead to apoptosis [10]. However, the cytotoxic effect of hIAPP and the mechanism of β-cell death are not yet well studied. A cell permeable Tat-CIAPIN1 protein was prepared for delivery of the CIAPIN1 protein into cells and tissue. Previous reports have shown that PTD fusion proteins can be used as tools for therapeutic protein application [23,24,25,26,40,41]. In the present study, we investigated whether Tat-CIAPIN1 protein could protect against cytotoxic effects of hIAPP, in vitro β-cell and in vivo T2DM animal model, and we observed that this fusion protein protected against hIAPP-induced cell death in RINm5F cell, as well as in pancreatic β-cell of the HFD combined with STZ-induced T2DM mouse model. Our previous studies have demonstrated that Tat-CIAPIN1 protein delivered into hippocampal neuronal cells can protect cells against death from oxidative stress and this protein inhibited cell death from cytokine-induced cytotoxicity in RINm5F cells [22,42]. Other studies have shown that ROS are important mediators of β-cell death associated with the pathogenesis of DM and that hIAPP-induced cytotoxicity leads to oxidative stress and cell death [43,44,45,46]. It is well known that oxidative stress is an important cause of diabetes and its associated complications. Additionally, the increased amount of ROS levels induced by oxidative stress serves an important role in the pathogenesis of diabetes [47,48]. Therefore we confirmed whether CIAPIN1 could inhibit hIAPP-induced cytotoxicity. In this study, we observed that hIAPP markedly produced ROS and DNA damage, whereas delivered Tat-CIAPIN1 protein significantly reduced hIAPP-induced cytotoxicity, indicating that Tat-CIAPIN1 protein could prevent hIAPP-induced cell death and plays cytoprotective roles in RINm5F cells.

It has been reported that oxidative stress is detrimental to many cells including pancreatic islets and that it can induce activation of MAPK signaling and activating of p38 and JNK MAP kinase signaling are one of the major T2DM risk factors [38,49]. Subramanian et al. have shown that JNK signaling is activated during islet amyloid formation and anti-apoptotic molecule Bcl-2 is increased in a JNK-dependent manner in hIAPP transgenic islets [38]. Furthermore, Park et al. have shown that CIAPIN1 can prevent the death of neurons by increasing Bcl-XL under oxidative stress, suggesting that CIAPIN1 has the function of protective effects on oxidative stress-induced apoptosis [21]. Other reports also showed that overexpression of CIAPIN1 can regulate cleaved Caspase-3/Caspase-3 expression in knock-down CIAPIN1 K562 cells [50,51]. Consistent with those results, we observed that MAPKs were activated and Bcl-2 expression was reduced in pancreatic β-cells treated with hIAPP. However, Tat-CIAPIN1 protein inhibited hIAPP-induced MAPK activation and increased Bcl-2 expression levels, indicating that Tat-CIAPIN1 protein could regulate MAPK activation with an anti-apoptotic function in hIAPP-induced pancreatic β-cells.

Diabetes is a chronic metabolic disease characterized by high blood glucose and it is well known that HFD feeding combined with a single or multiple intraperitoneal injection of low dose of STZ can successfully establish an experimental T2DM animal model [52,53]. In this study, a T2DM mouse model was established by using HFD combined with STZ to investigate effects of Tat-CIAPIN1 protein and we observed that blood glucose, body weight, and HbAlc levels were markedly increased in T2DM mice whereas Tat-CIAPIN1 protein reduced blood glucose, body weight, and HbAlc levels. There are few studies on the protective effect of CIAPIN1 protein on T2DM. In this study, we revealed the protective efficacy of CIAPIN1 protein against T2DM for the first time using Tat-CIAPIN1 protein. Recently, it has been reported that the hallmark of T2DM is the reduction and loss of islet β-cells [54,55] and a licochalcone can effectively reduce blood glucose and alleviate the phenomenon of weight loss in T2DM mice using HFD combined with STZ [52]. In addition, several reports have shown protective effects against T2DM animal model as well as diabetic complications [56,57,58]. Furthermore, it is known that T2DM is characterized by chronic inflammation which disrupts glucose homeostasis [59,60]. Several reports have revealed that inflammatory molecules such as interleukin 6 (IL-6), tumor necrosis factor-α (TNF-α), and C-reactive protein (CRP) are elevated in individuals with T2DM and associated with insulin resistance [61,62,63]. In a previous study, we have demonstrated that Tat-CIAPIN1 exerts a protective role in the inflammatory response and this protein markedly reduced the expression of IL-6 and TNF-α in lipopolysaccharide (LPS)-exposed Raw 264.7 cells and drastically decreased inflammation damage in a 12-O-tetradecanoylphorbol-13-acetate (TPA)-induced animal model [64]. Additionally, Tat-CIAPIN1 protein inhibits against cytokine-induced cytotoxicity in pancreatic RINm5F β-cells [42]. Oxidative stress caused by increased ROS is considered an important contributor to diabetes. Houstis et al. showed that increased ROS level is an important factor for insulin resistance because oxidative stress and hyperglycemia in T2DM triggers increasing of ROS. Thus, they suggested that antioxidant therapy might be a useful strategy in T2DM [65]. In this study, we showed that Tat-CIAPIN1 protein significantly reduced hIAPP-induced ROS generation in RINm5F cells. Therefore, we speculate that this fusion protein may play an important role in alleviating and protecting T2DM. Although more research is needed, in this study we observed that islet β-cells were unevenly destructed in the HFD + STZ group and this damage was protected by Tat-CIAPIN1 protein, indicating that CIAPIN1 can be beneficial for treatment of T2DM.

Further study is needed including validation of the Tat-CIAPIN1 protein for a signaling pathway in β-cells and T2DM animal model. Additionally, our findings raise issues that require further investigation. First, what are the downstream pathways when ROS and inflammation levels are elevated in T2DM? Since ROS and inflammation have been shown to induce various signaling pathways, the signaling pathways remain to be studied for further verification. Other studies showed that MAPK (JNK) is activated by oxidative stress and inhibition of JNK activation improves T2DM in mice [66,67]. Similarly, we showed that MAPK is activated in response to hIAPP, and that this effect is reversed by Tat-CIAPIN1 protein. Second, what is the cause of T2DM? The pathology of T2DM includes various factors such as aging, inflammation, and oxidative stress. Additionally, impaired muscle conditions may contribute to the development and progression of T2DM. Therefore, a study of precise function of the Tat-CIAPIN1 protein connected with various factors in T2DM is needed. Finally, further research on the development of disease treatment using cell permeable PTD fusion proteins is required. Delivery of therapeutic agents is a key point in the development of effective therapeutic agents for the treatment of various diseases including T2DM. To improve protein delivery efficacy, further studies are needed to find out the optimum condition of transduction because the efficacy of protein delivery depends on various factors including the type of PTD, the size of target protein, and cell types.

In summary, this study demonstrated that cell permeable Tat-CIAPIN1 protein inhibits hIAPP-induced pancreatic β-cells damage by suppressing hIAPP-induced cytotoxicity, MAPK, and apoptosis signaling pathways. Pretreatment of Tat-CIAPIN1 protein also attenuated diabetic damage by reducing blood glucose and HbAlc levels in a T2DM mouse model, suggesting that CIAPIN1 could be a useful potential therapeutic protein drug for T2DM.

## 4. Materials and Methods

### 4.1. Materials

Pancreatic β-cells (RINm5F β-cells) were obtained from the ATCC (Manassas, VA, USA). hIAPP (Sigma-Aldrich, St. Louise, MO, USA) was dissolved in dimethylsulfoxide. Fetal bovine serum (FBS) and 1% antibiotics (penicillin and streptomycin) were purchased from Gibco (Carlsbad, CA, USA). The used primary antibodies were obtained from Santa Cruz Biotechnology (Santa Cruz, CA, USA) and Cell Signaling Technology (Beverly, MA, USA). 2‘,7‘-Dichlorofluorescein diacetate (DCF-DA) was purchased from Sigma-Aldrich (St. Louis, MO, USA). All other agents were of the highest grade available unless otherwise stated.

### 4.2. Purification of Tat-CIAPIN1 Protein

Tat-CIAPIN1 protein was prepared as described previously [22]. To obtain Tat-CIAPIN1 and CIAPIN1 protein, the cDNA for human CIAPIN1 was amplified by PCR and the product was cloned into Tat expression vector. CIAPIN1 protein, without the Tat peptide, was also prepared to use as a control. Then, the Tat-CIAPIN1 and CIAPIN1 protein was expressed in Escherichia coli BL21 (DE3) cells by adding 0.5 mM isopropyl-β-D-thiogalactoside (Duchefa, Haarlem, Netherlands). Subsequently, Tat-CIAPIN1 protein was purified by a Ni^2+^-nitrilotri-acetic acid Sepharose affinity column (Qiagen, Valencia, CA, USA) and PD-10 column chromatography (Amersham, Braunschweig, Germany) according to the manufacturer’s instructions. Purified Tat-CIAPIN1 and CIAPIN1 protein concentration was determined by the Bradford assay [68].

### 4.3. Cell Culture and Delivery of Tat-CIAPIN1 Protein into RINm5F Cells

RINm5F cells were maintained in RPMI1640 medium containing 10% FBS and 1% antibiotics as described previously [42].

The intracellular delivery of Tat-CIAPIN1 protein in RINm5F cells were detected by confocal fluorescence microscopy as described previously [22]. Briefly, RINm5F cells were treated with Tat-CIAPIN1 (3 μM) protein for 1 h and fixed with 4% paraformaldehyde. Then, the cells were incubated with the histidine primary antibody and the Alexa fluor 488-conjugated secondary antibody. Nuclei were stained with DAPI (1 μg/mL; Roche Applied Science, Mannheim, Germany). Images were taken using confocal fluorescence microscopy using a model FV-300 microscope (Olympus, Tokyo, Japan).

The cells were treated with various concentrations of Tat-CIAPIN1 protein for 1 h or with time periods of Tat-CIAPIN1 protein. Subsequently, the cells were treated with trypsin-EDTA (Gibco, Grand Island, NY, USA) and washed twice with phosphate-buffered saline (PBS) and Western blotting was performed to determine delivered Tat-CIAPIN1 protein into the cells.

### 4.4. Cell Viability Assay

The effects of Tat-CIAPIN1 protein against hIAPP-induced cell death were determined by 3-(4,5-dimethyl-2-thiazolyl)-2,5-diphenyltetrazolium bromide (MTT) assay as described previously [69,70]. Briefly, RINm5F cells were plated onto 96-well plate for 24 h and Tat-CIAPIN1, CIAPIN1, or Tat peptide (3 μM) were added to the culture medium for 1 h. Subsequently, the cells were treated with 20 μM hIAPP for 24 h. Then, the absorbance was measured at 450 nm using an ELISA microplate reader (Multiskan MCC/340; Thermo Labsystems Oy., Helsinki, Finland) and the cell viability was defined as the % of untreated control cells.

### 4.5. Western Blot Analysis

Total protein was extracted from RINm5F cells and western blotting was performed as described previously [69,71]. Equal amounts of proteins were loaded into 12% SDS-PAGE and electrotransferred to a polyvinylidene difluoride (PVDF) membrane. The membrane was blocked with a TBS-T (25 mM Tris-HCl, 140 mM NaCl, 0.1% Tween 20, pH 7.5) buffer containing 5% non-fat dry milk for 1 h. After being washed with TBS-T buffer, the membrane was incubated with primary antibody followed by appropriate horseradish peroxidase-conjugated secondary antibody. Then, the membranes were washed with TBS-T buffer three times and the protein bands were identified using chemiluminescent reagents as recommended by the manufacturer (Amersham, Franklin Lakes, NJ, USA). The bands were quantified by Image J software (software version 1.45s; NIH, Bethesda, MD, USA).

### 4.6. Measurement of ROS Levels

Intracellular ROS levels were determined using 2′,7′-Dichlorofluorescein diacetate (DCF-DA) as described previously [69,72]. To examine intracellular ROS levels, RINm5F cells were treated with Tat-CIAPIN1, CIAPIN1, or Tat peptide (3 μM) for 1 h and exposed to hIAPP (20 μM) for 3 h. Then, the cells were washed with PBS and incubated for 30 min with DCF-DA (20 μΜ). Then, fluorescent images were obtained by fluorescence microscopy (Nikon eclipse 80i, Tokyo, Japan) and the fluorescence intensity was detected with excitation at 485 nm and emission at 538 nm using a Fluoroskan ELISA plate reader (Labsystems Oy, Helsinki, Finland).

### 4.7. TUNEL Assay

To examine whether Tat-CIAPIN1 protein protects against hIAPP-induced DNA damage in cells, RINm5F cells were pretreated with Tat-CIAPIN1, CIAPIN1, or Tat peptide (3 μM) for 1 h after which hIAPP (20 μM) was added to the culture medium for 3 h. Terminal deoxynucleotidyl transferase-mediated biotinylated dUTP nick end labeling (TUNEL) staining was performed using a Cell Death Detection kit (Roche Applied Science, Basel, Switzerland). Fluorescence images were obtained by fluorescence microscope (Nikon eclipse 80i, Tokyo, Japan). Fluorescence intensity levels were measured using a Fluoroskan ELISA plate reader (Labsystems Oy, Helsinki, Finland) at 485 nm excitation and 538 nm emission [22,69].

### 4.8. Animal Model and Treatments

Eight-week-old, male C57BL/6 mice were acquired from the Hallym University Experimental Animal Center. They were housed at 23 °C and humidity of 60%. They were exposed to regular 12 h cycles of light and dark and were given ad libitum access to food and water. All experimental procedures involving animals and their care conformed to the Guide for the Care and Use of Laboratory Animals of the National Veterinary Research and Quarantine Service of Korea and were approved by the Hallym Medical Center Institutional Animal Care and Use Committee (Hallym 2017-18). All animal experiments were performed according to the ARRIVE guideline (https://www.nc3rs.org.uk/arrive-guidelines, accessed on 14 February 2023).

High-fat diet (HFD) and streptozotocin (STZ)-induced diabetes model was prepared as described previously [52,73]. Male C57BL/6 mice were adaptively fed for 1 week, and then the mice were randomly divided into five groups (n = 7/each group). Group 1 (control) was maintained on a standard. Group 2–5 were allowed 6 weeks of free access to a HFD diet and given a single intraperitoneal injection of 60 mg/kg STZ to induce T2DM. The T2DM mice were randomly divided into the following groups: HFD/STZ, HFD/STZ + Tat-CIAPIN1, HFD/STZ + CIAPIN1, and HFD/STZ + Tat peptide-treated model. These mice received three injections of Tat-CIAPIN1 protein (2 mg/kg), CIAPIN1 protein (2 mg/kg), or Tat peptide (2 mg/kg) at 1, 3, and 5 days. After 8 weeks, mice were sacrificed and pancreatic tissues were removed for histological examinations. Pancreatic tissue sections were stained with insulin and hematoxylin and eosin (HE) as previously described [74,75].

Changes in blood glucose levels were analyzed using Accu-Chek glucose strips and Accu-Chek compact plus meter (Roche, Germany). To examine changes to blood glycated hemoglobin A1c (HbA1c) levels, blood samples were collected from the tail vein. HbA1c levels were then measured using a glycohemoglobin analyzer (HLCr-723GHb; Tosoh Corp., Kyoto, Japan).

### 4.9. Statistical Analysis

All data were represented as mean ± standard error of the mean. Student’s *t*-test and one-way ANOVA followed by a Bonferroni’s post-hoc test were performed for comparisons between two groups and for multiple comparisons, respectively. Difference at *p* < 0.05 was considered statistically significant.

## Data Availability

Data will be made available on request.
